# Healthcare Resource Utilization and Costs in Patients With Chronic Lymphocytic Leukemia or Small Lymphocytic Leukemia Treated With Covalent Bruton's Tyrosine Kinase Inhibitors: Real‐World Impact of Cardiovascular Adverse Events

**DOI:** 10.1002/cam4.71484

**Published:** 2025-12-21

**Authors:** Mavis Obeng‐Kusi, Enrico De Nigris, Siyang Leng, Mohammed Z. H. Farooqui, Halit O. Yapici, Ian Weimer, Weiqi Jiao, Hayden W. Hyatt, Xuan Zhang, Kunal Lodaya, David Dingli

**Affiliations:** ^1^ Merck & Co., Inc. Rahway New Jersey USA; ^2^ MSD (UK) Limited London UK; ^3^ Boston Strategic Partners, Inc. Boston Massachusetts USA; ^4^ Mayo Clinic Rochester Minnesota USA

**Keywords:** epidemiology, leukemia, target therapy, tyrosine kinase inhibitors

## Abstract

**Introduction:**

Although covalent Bruton's tyrosine kinase inhibitors (cBTKis) have transformed treatment of chronic lymphocytic leukemia and small lymphocytic leukemia (CLL/SLL), cBTKi‐related cardiotoxicity is a known side effect. This real‐world study evaluated incident cardiovascular adverse events (CVAE), healthcare resource utilization (HCRU), and costs among patients with CLL/SLL receiving cBTKis.

**Methods:**

Adult patients with CLL/SLL who initiated ibrutinib or acalabrutinib treatment (index date) between 2020 and 2023 were identified using US claims data and followed up to 36 months post‐index. Incident CVAEs (not present pre‐index) were assessed during cBTKi treatment and patients were stratified by CVAE status. HCRU and costs were evaluated per 1000 patient‐months (PPPM).

**Results:**

Overall, 2069 patients were identified (mean age of 73.7 ± 9.1 years, 40.4% female) with a mean treatment‐specific observation period of 11.4 ± 9.5 months. At least one incident CVAE was observed in 442 (21.4%) patients. The incidence rate was 21.6 PPPM for hypertension, 8.9 PPPM for atrial fibrillation, 8.6 PPPM for ventricular arrhythmias, and 8.3 PPPM for atrial flutter. Patients with incident CVAEs had significantly more total medical service days PPPM (4334 vs. 3138, *p* < 0.001), primarily driven by increased inpatient (690 vs. 230), outpatient (2798 vs. 2425), other visit (662 vs. 375), and ER days (184 vs. 109) than those without CVAEs. Total healthcare costs (PPPM) were substantially higher for patients with incident CVAEs ($20,250,560 vs. $17,413,460, *p* < 0.001) mainly due to higher inpatient ($3,417,948 vs. $915,839, *p* < 0.001), outpatient ($2,415,060 vs. $1,769,628, *p* < 0.01), and ER costs ($186,820 vs. $87,585, *p* < 0.001).

**Conclusions:**

The observed class‐effect of high CVAEs with cBTKis underscores the unmet need for safer, more selective agents for CLL/SLL treatment. The HCRU and economic burden remain high for CLL/SLL, especially among patients experiencing CVAEs during cBTKi treatment. These findings emphasize the importance of optimizing clinical management to reduce CVAE risk and downstream impacts on HCRU and costs.

## 1 Introduction

Chronic lymphocytic leukemia (CLL) is one of the most common types of leukemia, with > 215,000 individuals currently living with this disease in the United States [1]. Covalent Bruton's tyrosine kinase inhibitors (cBTKis)—including first‐generation ibrutinib and second‐generation agents such as acalabrutinib and zanubrutinib—have revolutionized the treatment landscape for CLL/small lymphocytic lymphoma (SLL) and have become standard of care for patients due to their demonstrated efficacy and profound improvements in progression‐free survival [2, 3, 4]. However, the use of cBTKis is not without risk. Cardiovascular toxicity from both on‐target and off‐target effects of cBTKis has emerged as a noteworthy concern, with prior randomized clinical trials revealing significant incidences of cardiovascular adverse events (CVAEs) [2, 3, 5].

The development of treatment‐emergent CVAEs associated with cBTKis, such as atrial fibrillation, hypertension, or ventricular arrhythmias, introduces additional complexity for treating patients with CLL/SLL [6]. While findings from clinical trials have indicated lower risk of CVAEs with second‐generation compared to first‐generation cBTKis, there is an established cBTKi class effect of an increased risk of CVAEs [4, 7, 8]. Patients experiencing CVAEs may require dose modifications or treatment discontinuation of cBTKis, further complicating care pathways and clinical outcomes [9]. Moreover, these events often necessitate frequent monitoring, concomitant medication use (e.g., anticoagulants), and, in many cases, inpatient care, contributing to increased healthcare resource utilization (HCRU) and economic burden [6, 10, 11]. This is an important consideration, given that the continued growth in United States (US) healthcare expenditures, particularly in oncology, has intensified the focus on value‐based care. National cancer‐related healthcare costs are projected to have risen from $182 billion in 2015 to over $222 billion in 2025 [12, 13, 14, 15]. Hence, understanding the full spectrum of HCRU and costs associated with effective cancer therapies, including those related to adverse event management (e.g., CVAEs), is essential for informing treatment selection, payer policy, and clinical decision‐making.

Real‐world data examining the downstream HCRU and economic impact of cBTKi‐related CVAEs remain scarce. Additionally, findings on CVAE incidence rates from previous clinical trials may be limited in their generalizability to real‐world practice due to rigid eligibility criteria [2, 16]. To address these gaps, the current study evaluated the real‐world incidence rate of CVAEs and their downstream impact on HCRU and costs among patients with CLL/SLL receiving cBTKi treatment. Using a large administrative claims database, this analysis aims to provide contemporary, generalizable insights into the clinical and economic burden associated with CVAEs during treatment with cBTKis among patients with CLL/SLL.

## 2 Methods

### 2.1 Data Source

Claims data from Optum's de‐identified Clinformatics Data Mart (CDM) database were evaluated to determine HCRU and healthcare costs among patients with CLL/SLL initiating treatment with ibrutinib or acalabrutinib between January 1, 2020, and January 1, 2023. Optum CDM is derived from a database of administrative health claims for members of large commercial and Medicare Advantage health plans. This study was approved for exemption under 45 CFR § 46.104(d) [4] from WIRB‐Copernicus Group Institutional Review Board (IRB).

### 2.2 Study Population

Adult patients (≥ 18 years of age) diagnosed with CLL or SLL who initiated acalabrutinib or ibrutinib treatment were included in the study. Patients receiving treatment with zanubrutinib were not assessed due to the proximity to the end of the study's observation period and the U.S. Food and Drug Administration (FDA) approval in January 2023 for treating patients with CLL/SLL. Patient's CLL/SLL diagnosis was confirmed via two diagnostic claims on distinct dates after January 1, 2013, using International Classification of Disease Ninth/Tenth Revision (ICD‐9, ICD‐10) codes (Table S1). The index date was the initiation of ibrutinib or acalabrutinib between January 1, 2020, and January 1, 2023, based on the date of dispensing from pharmacy. This index period was selected to provide contemporary real‐world evidence reflecting the availability and use of both first‐ and second‐generation cBTKis (ibrutinib and acalabrutinib) in routine clinical practice following FDA approval of acalabrutinib in December 2019. Patients were required to have continuous enrollment for at least 6 months pre‐ and 3 months post‐index date. Patients were excluded if they had claims for another cBTKi‐related malignancy, metastatic tumors, CLL/SLL‐related anticancer therapy (e.g., chemotherapy and immunotherapy), stem cell transplantation prior to their first CLL/SLL diagnosis, or patients with ≥ 1 pharmacy claim for a cBTKi prior to January 1, 2020 (Tables S2–S6).

Demographic and baseline clinical characteristics were assessed during the baseline period (i.e., 6 months) prior to or on the index date. Demographic information included age, sex, race, year of the CLL/SLL diagnosis and index date, geographic region, and insurance type. Clinical characteristics included index treatment, time from cancer diagnosis to BTKi initiation, National Cancer Institute (NCI) Comorbidity Index, and cardiovascular disease present during the six‐month baseline [17].

### 2.3 Incident CVAEs

To understand the impact of incident CVAEs on HCRU and healthcare cost outcomes, patients were stratified into those who experienced incident CVAEs (for each respective CVAE assessed and total incident CVAEs) and those who did not experience incident CVAEs. Incident CVAEs were determined utilizing relevant ICD‐9‐CM and ICD‐10‐CM codes, including atrial fibrillation, atrial flutter, ventricular arrhythmias, heart failure, hypertension, cardiac‐related death, conduction disorders, cardiomyopathy, and myocardial infarction (Table S7). Those who experienced atrial flutter were further stratified by codes designating typical/atypical atrial flutter and unspecified atrial flutter only. A similar approach was taken for patients who experienced ventricular arrhythmias stratified by codes designating premature ventricular contractions (PVC) only and those with non‐PVC codes. To be considered an incident CVAE, the CVAE of interest was only captured if it occurred during treatment exposure of index cBTKi and was not existent in the entire medical history for a patient within the Optum CDM database prior to index treatment. Patients with a documented history of a given CVAE before the index date were excluded from analyses of that specific CVAE type to ensure that only treatment‐emergent events (those developing during cBTKi exposure) were captured. The impact of line of therapy status at index (i.e., first line [1 L] and second‐line or later [2 L+]) on incident CVAEs was also explored.

### 2.4 HCRU and Healthcare Cost Outcomes

The total all‐cause HCRU and healthcare costs were captured for patients following initiation of cBTKis within the respective treatment period. The index cBTKi treatment‐specific observation period was defined as the period from the index date (i.e., initiation of cBTKi) to the earliest date of treatment discontinuation (the last day of supply before a gap of at least 90 consecutive days without another claim for the BTKi initiated on the index date), date before a change in treatment (the day before the initiation date of a therapeutic agent other than the BTKi initiated on the index date), or the end of data availability. HCRU was determined as the number of days with medical services, and costs were adjusted to 2023 USD using US dollars according to the medical care and prescription drug component of the Consumer Price Index. All‐cause HCRU and costs were further broken down by place of care service (based on unique claims occurring on separate days): inpatient, outpatient, emergency, and other services. Other services included home services and hospice. In addition, costs associated with pharmacy claims were also captured. HCRU and costs were described on a per 1000 patient‐month basis.

### 2.5 Statistical Analysis

Patient characteristics were summarized descriptively using means (SDs) and medians (IQRs) or frequencies and proportions. A Chi‐square test was used to compare descriptive result differences between cohorts. HCRU rate differences between those who did or did not experience any incident cBTKi‐related CVAE of interest were analyzed with Z tests to generate P‐values and rate ratios (95% CIs). Cost rate differences were compared between those who did or did not experience any incident cBTKi‐related CVAE of interest using univariate Gamma general linear models (GLMs) to generate P‐values and cost ratios (95% CIs). Descriptive statistics were provided for further breakdown of HCRU and costs among each of the CVAE types assessed.

## 3 Results

Overall, 2069 eligible patients were identified with a mean ± SD age of 73.7 ± 9.1 years, and 40.4% were female (Table 1). The mean ± SD (median) treatment‐specific observation period was 11.4 ± 9.5 (8.5) months. The majority of patients had Medicare Advantage coverage (79.8%), with 20.2% having commercial insurance.

**TABLE 1:** Patient demographics and baseline characteristics.

Baseline characteristics	Overall (*N* = 2069)	Patients with ≥ 1 incident CVAE (*N* = 442)	Patients without incident CVAE (*N* = 1627)
Treatment‐specific observation period^a^, months, mean ± SD [median]	11.4 ± 9.5 [8.5]	13.5 ± 9.9 [11.0]	10.8 ± 9.3 [7.9]
Age, years, mean ± SD [median]	73.7 ± 9.1 [74.0]	74.8 ± 8.4 [75.0]	73.4 ± 9.3 [74.0]
Race, *n* (%)			
White	1548 (74.8%)	334 (75.6%)	1214 (74.6%)
Black	230 (11.1%)	46 (10.4%)	184 (11.3%)
Asian	36 (1.7%)	9 (2.0%)	27 (1.7%)
Hispanic	117 (5.7%)	21 (4.8%)	96 (5.9%)
Unknown	139 (6.7%)	32 (7.2%)	106 (6.5%)
Year of index date^b^, *n* (%)			
2020	704 (34.0%)	177 (40.0%)	527 (32.4%)
2021	784 (37.9%)	170 (38.5%)	613 (37.7%)
2022	582 (28.1%)	95 (21.5%)	487 (29.9%)
Index cBTKi			
Acalabrutinib	868 (42.0%)	117 (26.5%)	751 (46.2%)
Ibrutinib	1201 (58.0%)	325 (73.5%)	876 (53.8%)
Geographic region^b^, ^c^, *n* (%)			
South	804 (38.8%)	161 (36.4%)	643 (39.5%)
West	530 (25.6%)	117 (26.5%)	413 (25.4%)
Midwest	487 (23.5%)	112 (25.3%)	375 (23.0%)
Northeast	248 (12.0%)	52 (11.8%)	196 (12.0%)
Insurance plan type^b^, ^c^, *n* (%)			
Medicare advantage	1651 (79.8%)	371 (83.9%)	1280 (78.7%)
*Commercial* *insurance*	418 (20.2%)	71 (16.1%)	347 (21.3%)
*POS*	188 (9.1%)	25 (5.7%)	163 (10.0%)
*HMO*	77 (3.7%)	13 (2.9%)	64 (3.9%)
*EPO*	58 (2.8%)	10 (2.3%)	48 (3.0%)
*IND*	26 (1.3%)	5 (1.1%)	21 (1.3%)
*OTH*	66 (3.2%)	17 (3.8%)	49 (3.0%)
Time from CLL/SLL diagnosis to BTKi initiation, months, mean ± SD [median]	30 ± 29 [20]	28 ± 29 [17]	30 ± 29 [21]
NCI^d^, mean ± SD [median]	0.5 ± 0.5 [0.5]	0.5 ± 0.5 [0.5]	0.5 ± 0.5 [0.5]
Baseline cardiovascular disease^d^			
Hypertension	1320 (63.8%)	277 (62.7%)	1043 (64.1%)
Ventricular arrhythmias^e^	276 (13.3%)	61 (13.8%)	215 (13.2%)
Heart failure	*261* (*12.6*%)	57 (12.9%)	204 (12.5%)
Atrial fibrillation	238 (11.5%)	*52* (11.8%)	*186* (11.4%)
Atrial flutter^f^	187 (9.0%)	35 (7.9%)	152 (9.3%)
Myocardial Infarction	106 (5.1%)	19 (4.3%)	87 (5.3%)
Cardiomyopathy	51 (2.5%)	16 (3.6%)	35 (2.2%)
Conduction disorders	12 (0.6%)	< 5 (< 1%)	11 (0.7%)

Abbreviations: BTKi, bruton tyrosine kinase inhibitor; cBTKI, covalent Bruton's tyrosine kinase inhibitors; CLL, chronic lymphocytic leukemia; CVAE, cardiovascular adverse event; EPO, Exclusive Provider Organization; HMO, Health Maintenance Organization; IND, individual plan; NCI, National Cancer Institute Comorbidity Index; OTH, other insurance type; POS, point of service; SD, standard deviation; SLL, small lymphocytic lymphoma.

^a^ The observation period spanned from the index date to the earliest of treatment discontinuation, date of death, end of continuous enrollment, or end of data availability.

^b^ Evaluated at the index date.

^c^ Geographic Region classified as ‘unknown’ and insurance plan type classified as PPO or missing only occurred in < 5 (< 1%) of evaluated patients.

^d^ Evaluated during the 6‐month baseline period.

^e^ Includes PVC‐only and non‐PVC ventricular arrhythmias.

^f^ Includes typical and atypical atrial flutter; missing and unspecified atrial flutter was not detected at baseline.

Of the 2069 patients, the most frequent incident CVAEs assessed were hypertension (21.4%; 21.6 per 1000 patient‐months), atrial fibrillation (9.8%; 8.9 per 1000 patient‐months), ventricular arrhythmias (9.6%; 8.6 per 1000 patient‐months), and atrial flutter (9.2%; 8.3 per 1000 patient‐months) (Table 2). Other notable CVAEs included heart failure (8.0%; 7.1 per 1000 patient‐months) and myocardial infarction (3.6%; 3.2 per 1000 patient‐months). Cardiac‐related death occurred in 1.2% of patients (1.0 per 1000 patient‐months). Incident CVAEs stratified by line of therapy status are reported in Table S8, with 21.2% of 1 L patients experiencing ≥ 1 incident CVAE and 22.2% for 2 L+ patients.

**TABLE 2:** Occurrence of incident CVAEs while being treated with cBTKis.

CVAE^a^	Overall (*N* = 2069)
Atrial fibrillation	*N* = 1750
Occurrence, *n* (%)	171 (9.8%)
Occurrence rate, PPPM	8.9
Atrial flutter	*N* = 1784
Occurrence, *n* (%)	164 (9.2%)
*Typical/atypical*	72 (4.0%)
*Unspecified atrial flutter only*	92 (5.2%)
Occurrence rate, PPPM	8.3
Heart failure	*N* = 1663
Occurrence, *n* (%)	133 (8.0%)
Occurrence rate, PPPM	7.1
Ventricular arrhythmias	*N* = 1603
Occurrence, *n* (%)	154 (9.6%)
*Non‐PVC‐related*	42 (2.6%)
*PVC‐only*	112 (7.0%)
Occurrence rate, PPPM	8.6
Hypertension	*N* = 444
Occurrence, *n* (%)	95 (21.4%)
Occurrence rate, PPPM	21.6
Cardiac‐related death	*N* = 2033
Occurrence, *n* (%)	24 (1.2%)
Occurrence rate, PPPM	1.0
Conduction disorders	*N* = 2026
Occurrence, *n* (%)	8 (0.4%)
Occurrence rate, PPPM	0.3
Cardiomyopathy	*N* = 1951
Occurrence, *n* (%)	48 (2.5%)
Occurrence rate, PPPM	2.2
Myocardial infarction	*N* = 1834
Occurrence, *n* (%)	66 (3.6%)
Occurrence rate, PPPM	3.2

Abbreviations: CVAE, cardiovascular adverse event; PPPM, per 1000 patient months; PVC, premature ventricular contraction.

^a^ The percentage for each CVAE occurrence is calculated relative to the total number of patients who did not have that specific CVAE in their entire medical history within the Optum CDM database (indicated by the *N*‐value for each CVAE type).

**FIGURE 1:**
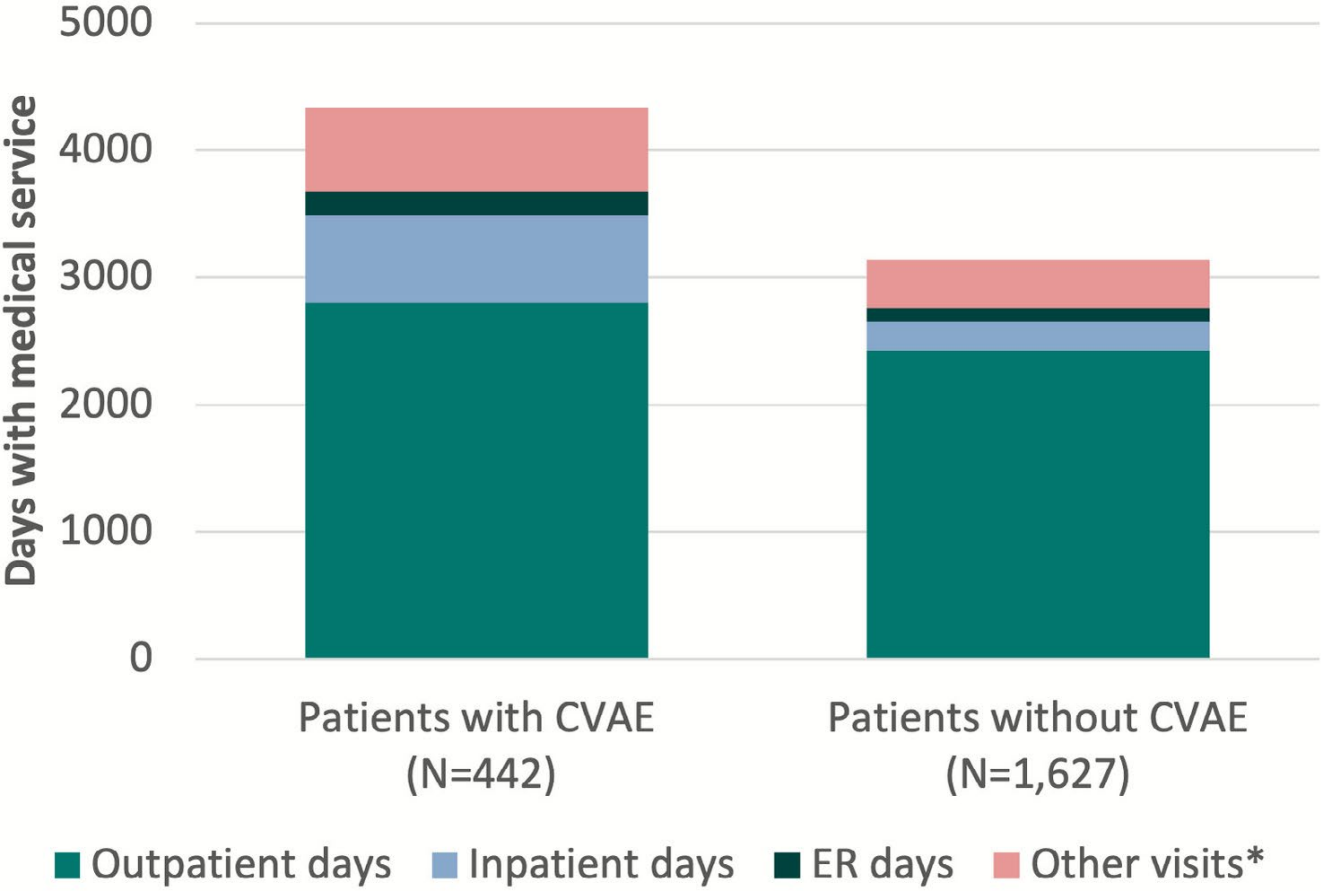
HCRU per 1000 patient‐months stratified by those who experienced incident CVAEs. ^†^Includes visits such as home services and hospice. CVAE, cardiovascular adverse event; ER, emergency room.

Patients who experienced a CVAE had a higher rate of total medical service days (4334 vs. 3138 days per 1000 patient‐months) (Figure 1). This corresponded to an all‐cause HCRU rate ratio (95% CI) of 1.38 (1.35, 1.41; *p* < 0.001) between patients who experienced a CVAE and those who did not. The higher HCRU observed in patients with an incident CVAE was driven by an increased number of inpatient (690 vs. 230) days with a rate ratio (95% CI) of 2.99 (2.89, 3.10; *p* < 0.001) and outpatient (2798 vs. 2425) days with a rate ratio of 1.15 (1.13, 1.18; *p* < 0.001).

Further breakdown by the specific CVAEs assessed revealed that patients who experienced conduction disorders had the highest rate difference (1241 more days with medical service on a per 1000 patient‐month basis) between those with the CVAE compared to those without, followed by heart failure (829 day difference), ventricular arrhythmias (794 day difference), myocardial infarction (703 day difference), atrial flutter (580 day difference), and atrial fibrillation (518 day difference) (Table 3). For the majority of CVAEs, inpatient visits/days were the largest driver in total HCRU differences between those with and without the specific CVAE of interest.

**TABLE 3:** All‐cause HCRU per 1000 patient‐months stratified by specific incident CVAE type.

CVAE (*n* with CVAE)	HCRU rate by place of service	Patients with CVAE	Patients without the CVAE	HCRU rate difference^c^
Ventricular arrhythmia (*n* = 133)	Total days with medical services	2152.9	1358.6	794.3
	*Inpatient* ^a^	478.7	101.0	377.7
	*Outpatient*	1260.1	1146.5	113.5
	*ER*	18.3	9.5	8.8
	*Other* ^b^	395.8	101.6	294.2
Atrial flutter (*n* = 164)	Total days with medical services	1970.9	1390.8	580.1
	*Inpatient* ^a^	413.0	122.1	291.0
	*Outpatient*	1225.6	1141.3	84.3
	*ER*	17.9	9.8	8.2
	*Other* ^b^	314.4	117.7	196.6
Atrial fibrillation (*n* = 171)	Total days with medical services	1911.0	1393.0	518.0
	*Inpatient* ^a^	405.6	121.8	405.6
	*Outpatient*	1213.3	1141.7	1213.3
	*ER*	17.4	9.8	17.4
	*Other* ^b^	274.7	119.8	274.7
Hypertension (*n* = 95)	Total days with medical services	1365.2	1316.4	48.8
	*Inpatient* ^a^	105.5	91.9	13.6
	*Outpatient*	1076.2	1136.5	−60.3
	*ER*	4.7	6.2	−1.5
	*Other* ^b^	178.9	81.9	97.0
Heart Failure (*n* = 154)	Total days with medical services	2172.1	1342.8	829.3
	*Inpatient* ^a^	447.4	98.2	349.2
	*Outpatient*	1286.5	1140.4	146.1
	*ER*	20.3	9.0	11.2
	*Other* ^b^	417.9	95.2	322.7
Myocardial infarction (*n* = 66)	Total days with medical services	2125.4	1422.9	702.5
	*Inpatient* ^a^	470.8	140.1	330.7
	*Outpatient*	1211.8	1155.2	56.5
	*ER*	17.2	10.6	6.6
	*Other* ^b^	425.7	117.0	308.7
Cardiomyopathy (*n* = 48)	Total days with medical services	2016.9	1464.4	552.5
	*Inpatient* ^a^	453.7	153.6	300.1
	*Outpatient*	1179.7	1165.4	14.3
	*ER*	8.6	11.1	−2.5
	*Other* ^b^	375.0	134.4	240.6
Cardiac‐related death (*n* = 24)	Total days with medical services	2282.7	1486.0	796.8
	*Inpatient* ^a^	668.1	163.4	504.8
	*Outpatient*	1270.1	1161.7	108.4
	*ER*	17.4	11.5	5.9
	*Other* ^b^	327.1	149.4	177.7
Conduction Disorders (*n* = 8)	Total days with medical services	2725.3	1483.7	1241.6
	*Inpatient* ^a^	993.1	158.0	835.1
	*Outpatient*	1331.8	1168.7	163.2
	*ER*	69.3	11.2	58.1
	*Other* ^b^	331.0	145.9	185.1

Abbreviations: CVAE, cardiovascular adverse event; ER, emergency room; HCRU, healthcare resource utilization.

^a^ Includes hospitalizations and skilled nursing facilities. Inpatient days were captured as unique claims on distinct dates, which could potentially cause an underestimation of the actual visits patients had.

^b^ Includes visits such as home services and hospice.

^c^ The comparator group for each specific CVAE is the cohort of assessed patients who did not experience that specific incident CVAE.

Total costs were higher in patients who experienced an incident CVAE compared to those who did not ($20,250,560 vs. $17,413,460 per 1000 patient‐months) resulting in a cost ratio (95% CI) of 1.25 (1.19, 1.31; *p* < 0.001) (Table 4). Inpatient costs ($3,417,948 vs. $915,839) were the primary driver in total cost differences between those with and without CVAEs, followed by outpatient costs ($2,415,060 vs. $1,769,628). Pharmacy costs contributed to the largest proportion of cost rates for both cohorts ($14,016,900 for those with a CVAE vs. $14,473,270 for those without).

**TABLE 4:** All‐cause healthcare costs per 1000 patient‐months stratified by patients experiencing an incident CVAE.

Cost rate by place of service, 2023 USD	Patients with any CVAE (*N* = 442)	Patients without any CVAE (*N* = 1627)	Cost difference between cohorts^c^	Cost ratio^c^ (95% CI)
Total medical and pharmacy costs	$20,250,560	$17,413,460	$2,837,100	1.248 (1.190, 1.308)*
*Inpatient* ^a^	$3,417,948	$915,839	$2,502,109	3.609 (2.670, 4.878)**
*Outpatient*	$2,415,060	$1,769,628	$645,432	1.183 (1.041, 1.344)*
*ER*	$186,820	$87,585	$99,235	2.869 (2.258, 3.646)*
*Other* ^b^	$213,836	$167,132	$46,704	1.251 (0.972, 1.610)
*Pharmacy*	$14,016,900	$14,473,270	‐$456,370	0.947 (0.915, 0.979)**

Abbreviations: CI, confidence interval; CVAE, cardiovascular adverse event; ER, emergency room.

* *p*‐value < 0.001.

** *p*‐value < 0.01.

^a^ Includes hospitalizations and skilled nursing facilities.

^b^ Includes visits such as home services and hospice.

^c^ The comparator group for calculating the cost difference and cost ratio is the cohort of patients without any CVAEs.

Breakdown of costs by service types for each specific CVAE assessed are provided in Table 5. Patients who experienced incident atrial fibrillation had higher costs than those without atrial fibrillation ($3,789,820 million higher per 1000 patient‐months). The cost difference was also higher in those with the specific incident CVAE than those without for atrial flutter ($3,867,160 difference), ventricular arrhythmias ($3,674,860 difference), and hypertension ($721,840). For the majority of CVAEs, inpatient costs were the largest driver in total cost differences between those with and without the specific CVAE of interest.

**TABLE 5:** All‐cause healthcare costs per 1000 patient‐months stratified by specific incident CVAE type.

CVAE (*n* with CVAE)	Cost rate by place of service, 2023 USD	Patients with CVAE	Patients without the CVAE	Cost difference between cohorts^c^
Ventricular arrhythmia (*n* = 133)	Total medical + pharmacy	$21,084,000	$17,409,140	$3,674,860
	*Inpatient* ^a^	$4,198,416	$899,046	$3,299,371
	*Outpatient*	$2,733,373	$1,753,632	$979,741
	*ER*	$235,412	$66,878	$168,535
	*Other* ^b^	$295,423	$102,064	$193,359
	*Pharmacy*	$13,621,380	$14,587,520	‐$966,140
Atrial flutter (*n* = 164)	Total medical + pharmacy	$21,361,640	$17,494,480	$3,867,160
	*Inpatient* ^a^	$4,467,108	$1,030,195	$3,436,913
	*Outpatient*	$2,662,590	$1,734,666	$927,924
	*ER*	$198,840	$93,672	$105,168
	*Other* ^b^	$236,881	$162,377	$74,504
	*Pharmacy*	$13,796,220	$14,473,570	‐$677,350
Atrial fibrillation (*n* = 171)	Total medical + pharmacy	$21,291,880	$17,502,060	$3,789,820
	*Inpatient* ^a^	$4,270,567	$1,014,868	$3,255,699
	*Outpatient*	$2,685,874	$1,742,053	$943,821
	*ER*	$202,140	$93,742	$108,398
	*Other* ^b^	$228,190	$165,419	$62,771
	*Pharmacy*	$13,905,110	$14,485,980	‐$580,870
Hypertension (*n* = 95)	Total medical + pharmacy	$17,786,800	$17,064,960	$721,840
	*Inpatient* ^a^	$902,323	$702,566	$199,758
	*Outpatient*	$1,906,302	$1,844,124	$62,178
	*ER*	$79,758	$38,157	$41,602
	*Other* ^b^	$151,542	$79,813	$71,729
	*Pharmacy*	$14,746,870	$14,400,300	$346,570
Heart failure (*n* = 154)	Total medical + pharmacy	$21,327,550	$17,316,790	$4,010,760
	*Inpatient* ^a^	$3,964,977	$857,158	$3,107,819
	*Outpatient*	$2,685,380	$1,717,088	$968,292
	*ER*	$200,167	$66,345	$133,822
	*Other* ^b^	$288,326	$102,561	$185,765
	*Pharmacy*	$14,188,700	$14,573,640	‐$384,940
Myocardial infarction (*n* = 66)	Total medical + pharmacy	$23,789,660	$17,799,750	$5,989,910
	*Inpatient* ^a^	$6,915,976	$1,195,359	$5,720,617
	*Outpatient*	$2,823,915	$1,854,152	$969,763
	*ER*	$279,715	$98,171	$181,544
	*Other* ^b^	$230,068	$162,881	$67,188
	*Pharmacy*	$13,539,990	$14,489,190	‐$949,200
Cardiomyopathy (*n* = 48)	Total medical + pharmacy	$20,762,280	$17,912,950	$2,849,330
	*Inpatient* ^a^	$5,412,183	$1,337,941	$4,074,242
	*Outpatient*	$2,712,882	$1,878,979	$833,903
	*ER*	$261,615	$105,659	$155,956
	*Other* ^b^	$219,601	$169,972	$49,629
	*Pharmacy*	$12,155,990	$14,420,400	‐$2,264,410
Cardiac‐related death (*n* = 24)	Total medical + pharmacy	$28,578,960	$17,968,320	$10,610,640
	*Inpatient* ^a^	$9,547,705	$1,450,288	$8,097,417
	*Outpatient*	$2,931,861	$1,898,895	$1,032,966
	*ER*	$566,970	$107,143	$459,827
	*Other* ^b^	$162,251	$180,643	‐$18,392
	*Pharmacy*	$15,370,170	$14,331,350	$1,038,820
Conduction disorders (*n* = 8)	Total medical + pharmacy	$28,479,470	$17,937,680	$10,541,790
	*Inpatient* ^a^	$11,639,410	$1,432,343	$10,207,067
	*Outpatient*	$2,427,259	$1,892,459	$534,800
	*ER*	$439,868	$98,000	$341,868
	*Other* ^b^	$356,973	$144,575	$212,398
	*Pharmacy*	$13,615,960	$14,370,300	‐$754,340

Abbreviations: CVAE, cardiovascular adverse event; ER, emergency room.

^a^ Includes hospitalizations and skilled nursing facilities. Inpatient days were captured as unique claims on distinct dates, which could potentially cause an underestimation of the actual visits patients had.

^b^ Includes visits such as home services and hospice.

^c^ The comparator group for each specific CVAE is the cohort of assessed patients who did not experience that specific incident CVAE.

## 4 Discussion

Despite the profound clinical utility of cBTKi treatment for patients with CLL/SLL, incident CVAEs due to cBTKi‐related cardiovascular toxicity can have devastating consequences for patients and increase the toll of treatment management for healthcare systems. The current findings revealed that 21.4% of patients experienced an incident CVAE while receiving cBTKi treatment. Moreover, those who experienced a CVAE had substantially higher costs and HCRU than those without a CVAE, largely driven by increases in inpatient care. Collectively, this study provides insight into the occurrence of incident CVAEs and their economic impacts in over 2000 patients with CLL/SLL treated with cBTKis in real‐world settings across the United States.

While prior clinical trials have provided robust insight into the occurrence of CVAEs during cBTKi treatment, the generalizability of these findings may be limited due to strict recruitment criteria, such as exclusion of patients with significant pre‐existing cardiovascular disease [2, 16]. Our findings on the occurrence of incident CVAEs revealed directionally similar but higher rates than those reported in prior clinical trials. For instance, ventricular arrhythmias occurred in ≤ 1% of patients treated in clinical trials compared to the current findings with 2.6% of patients experiencing incident non‐PVC related ventricular arrhythmias and 7.0% developing incident PVCs [2, 3, 4]. Similarly, the incidence rates for atrial fibrillation and hypertension were also 2‐ to 3‐fold higher than prior reports for patients with CLL/SLL receiving cBTKis at 8.9 and 21.6 per 1000 patient‐months, respectively [4, 18]. Nonetheless, the current cohort was older than patients included in prior clinical trials (median age of 74 years compared to ~68 years), which may have also contributed to higher incidence rates for CVAEs given that age is a risk factor for the development of cardiovascular conditions. Importantly, prior epidemiological surveillance and real‐world evidence studies report similar patient demographics and patient age at diagnosis with CLL/SLL compared to the current study cohort, providing further support that these findings are representative of the cardiovascular disease burden in the general population of those treated in real‐world settings in the US [19, 20, 21].

While treatment‐emergent CVAEs are well‐recognized complications that may occur in patients treated with cBTKis [5, 6], this study provides novel insights into the HCRU and economic burden of incident CVAEs among patients with CLL/SLL receiving cBTKis. In this regard, patients with incident CVAEs required significantly greater HCRU and incurred higher healthcare costs, corresponding to a ~38% and ~25% higher total rate for HCRU and costs compared to those without incident CVAEs. For instance, the cost difference among those with and without an incident CVAE exceeded $3,600,000 on a per 1000 patient‐month basis for patients with atrial fibrillation, atrial flutter, and ventricular arrhythmias (higher costs in those with the CVAE). Moreover, the cost rate difference was approximately $6,000,000 higher for those with myocardial infarction and over $10,000,000 higher for conduction disorders and cardiac‐related death compared to those without these specific CVAEs. Cost and HCRU rate differences were the lowest for hypertension, likely reflecting the relatively low level of intervention typically required for its management (e.g., pharmacotherapy without hospitalization), in contrast to the more resource‐intensive treatment of other CVAEs. Overall, the largest driver for higher total costs in those with CVAEs was inpatient costs, which comprised 88% of the total cost difference and were more than 3‐fold higher for those with a CVAE compared to those without. Indeed, patients with at least one incident CVAE had over 450 more inpatient days on a per‐1000 patient month basis compared to those without a CVAE.

The substantial increase in costs and HCRU observed by the current findings reflect the multi‐factorial challenges posed by cBTKi‐related cardiovascular toxicity for both individual patients and the healthcare systems responsible for patient care. For instance, patients who develop atrial fibrillation often require hospitalization to closely monitor cardiovascular conditions and initiate treatment for atrial fibrillation, while also being at higher risk for re‐hospitalization, morbidity, and mortality [6, 11]. Regarding treatment management, incident atrial fibrillation increases the complexity of treatment regimens in order to mitigate the risk of atrial fibrillation‐associated complications (e.g., anticoagulant therapy to prevent thromboembolic events) and balancing risks associated with concomitant medications (e.g., combined risk of bleeding due to anticoagulants and cBTKi‐related platelet dysfunction) [6, 22]. These types of challenges are present for each of the CVAEs assessed, and management strategies often focus on early identification and intervention, including dose adjustments, lifestyle modifications, or switching to alternative cBTKis to mitigate risks and ensure continuity of treatment [23]. Therapeutic strategies for first‐line patients with elevated cardiovascular risk may also include weighing monotherapy with cBTKis against combination treatment, such as cBTKis with BCL2 inhibitors (e.g., venetoclax) [24, 25]. Overall, the decisions for treatment management of incident CVAEs must weigh the risk of CLL/SLL disease progression (e.g., in cases of switching or discontinuation of cBTKis) with the severity of the CVAE, often necessitating multidisciplinary collaboration to optimize patient outcomes [26].

Escalating healthcare costs, especially in oncology, have fueled ongoing debate about best practices to balance cost control with quality care [13]. While mixed evidence exists regarding the cost‐effectiveness of cBTKis [27, 28], the cardiovascular toxicity associated with current generation cBTKis remains a serious concern for clinicians and underscores the importance of evaluating both economic impact and clinical value. The current findings illustrate the importance of proactive safety monitoring during cBTKi treatment to detect early signs of cBTKi‐related cardiovascular toxicity and potentially reduce the need for more costly interventions later in the disease course. Moreover, the development and adoption of novel therapies with more favorable cardiovascular toxicity safety profiles could lead to HCRU and cost savings by reducing the incidence of CVAEs and their downstream care.

Notably, the costs and HCRU observed in the current findings are generally aligned with findings from prior studies in patients with CLL treated with ibrutinib. The proportion of costs attributed to the various places of service categories (e.g., pharmacy) is similar to prior findings among patients with CLL/SLL receiving cBTKis, supporting the rigor of the current methodology [10, 29, 30]. Overall costs were higher than the findings reported by Goyal et al. ($14,158 per patient per month converted to 2023 USD) and lower than findings from Kabadi et al. ($25,909 per patient per month converted to 2023 USD). The variations in cost reporting likely reflect underlying payer differences. Goyal et al. reported cost estimates derived from Medicare claims data, with the authors noting that their findings were lower than studies on patients with CLL enrolled in private insurance potentially due to differences in reimbursement rates [10]. In contrast, the findings reported in Kabadi et al. reflect employer‐sponsored private health insurance plans which may have had higher reimbursement rates [29]. Compared to Kabadi et al., the current findings reflect patients covered by a range of healthcare insurance plans but primarily consist of patients with Medicare Advantage coverage. Collectively, these findings provide the most up‐to‐date analysis of HCRU and costs among patients with CLL/SLL treated with cBTKis and provide insight into the burden of incident CVAEs in this patient population.

### 4.1 Limitations

All limitations regarding research conducted with secondary data apply to this study. For instance, the causality of incident CVAEs determined utilizing claims data cannot be established. The absence of a claim for a disease, medication, or procedure was assumed to indicate that the patient did not have the disease or received the corresponding treatment. The analysis did not adjust for baseline patient characteristics, and the unadjusted nature of the HCRU and cost comparisons means the reported differences reflect the total incremental healthcare system burden for patients with incident CVAEs, encompassing both the event management costs and underlying patient complexity. Hence, a small portion of the captured HCRU and costs may reflect management of co‐occurring conditions unrelated to CLL/SLL (e.g., non‐metastatic, non‐cBTKi‐related malignancies), reflecting the HCRU and economic burden that occurs in the real‐world setting. Given the known association between cBTKi treatment and cardiotoxicity, and because our approach assessed incident CVAEs, the findings reflect the differences in costs/HCRU for CVAEs associated with cBTKi treatment. In addition, comparisons between those with and without CVAEs are subject to immortal time bias. Our analyses do not offer granular reporting on the impact of various treatment and patient characteristics on costs and HCRU (e.g., concomitant medications, payer type, and severity of CVAEs assessed). Although the primary intent was to report HCRU and costs during cBTKi treatment, the use of a 90‐day window after the last dose prescribed would also capture HCRU costs incurred up to 90 days after cBTKi discontinuation for patients who stopped treatment. Nonetheless, this approach provides insights into HCRU and costs that may be associated with events leading to discontinuation among these patients (e.g., CVAE). In cases with fewer than 1000 patient‐months of observation available for low‐frequency CVAE types (e.g., conduction disorders), HCRU and cost values were extrapolated to a 1000 patient‐month basis to facilitate standardized, time‐adjusted comparisons across cohorts. This study provided insights on the overall cost impact of patients treated with ibrutinib and acalabrutinib; however, patients receiving treatment with zanubrutinib were not included in this study due to the limited data available given the short overlap between the end of the study's identification period for patient index (January 1, 2023) and FDA approval of zanubrutinib (January 2023). Lastly, this assessment focused solely on direct medical costs and did not account for indirect costs, such as lost productivity, time away from daily activities, and emotional burden, which may underestimate the full economic and societal impact of treatment.

## 5 Conclusions

These findings demonstrate the considerable cardiovascular disease burden associated with cBTKi treatment for patients with CLL/SLL. The HCRU and financial burden remain high, particularly for those who experience CVAEs during cBTKi treatment. Patients who experience incident CVAEs incur higher HCRU, including more inpatient hospitalization days, which contribute to substantially higher costs for these patients with CLL/SLL. These findings highlight the importance of careful treatment selection, guided by proactive cardiovascular risk assessment and monitoring, and the implementation of integrated cardio‐oncology approaches to minimize CVAE risk, improve patient outcomes, and reduce healthcare costs and resource utilization. The persistent cardiovascular toxicity associated with the current generation cBTKis underscores the ongoing unmet need for safer and more efficacious drugs in this setting.

## Author Contributions

Conceptualization: D.D., E.D.N, H.O.Y., M.O.‐K. Methodology: E.D.N, H.O.Y., M.O.‐K., S.L., K.L. Software: I.W., X.Z. Writing – original draft: M.O.‐K., H.W.H. Writing – review and editing: all authors. Data curation: I.W., W.J., X.Z. Investigation: H.O.Y., W.J., H.W.H. Resources: E.D.N, S.L., M.Z.H.F, M.O.‐K. Supervision: D.D., E.D.N, S.L., K.L., M.O.‐K. Validation: I.W., W.J., H.W.H., X.Z., H.O.Y.

## Funding

This study was sponsored by Merck Sharp & Dohme LLC, a subsidiary of Merck & Co. Inc., Rahway, NJ, USA (MSD). Writing and editorial support was provided by Boston Strategic Partners Inc., funded by MSD. The funder was involved in all aspects of the design and conduct of the study; collection, management, analysis, and interpretation of the data; preparation, review, and approval of the manuscript; and decision to submit the manuscript for publication.

## Ethics Statement

This study was approved for exemption under 45 CFR § 46.104(d) (4) from WIRB‐Copernicus Group Institutional Review Board (IRB). Only de‐identified data were utilized for this analysis; therefore, informed consent was not required.

## Conflicts of Interest

Dr. David Dingli reports consulting for Alexion, Apellis, BMS, Jannsen, MSD, Novartis, Sanofi, Sorrento, Regeneron, and Genentech, and receiving research funding from Apellis and K36 Therapeutics. Enrico De Nigris is an employee of MSD (UK) Limited, London, UK. Siyang Leng, Mohammed Z. H. Farooqui, and Mavis Obeng‐Kusi are employees of Merck Sharp & Dohme LLC, a subsidiary of Merck & Co. Inc., Rahway, NJ, USA. Dr. Halit Yapici, Ian Weimer, Weiqi Jiao, Hayden Hyatt, Dr. Xuan Zhang, and Dr. Kunal Lodaya are employees of Boston Strategic Partners, a company that has provided paid services to MSD.

## Supporting information


**Table S1:** List of ICD‐9‐CM and ICD‐10‐CM codes for CLL and SLL.
**Table S2:** List of anticancer systemic agents CLL‐ or SLL‐related.
**Table S3:** List of CPT codes for radiation therapies for CLL or SLL.
**Table S4:** List of HCPCS, CPT, and ICD‐10‐PCS Codes for CAR‐T‐Cell Therapy.
**Table S5:** List of HCPCS, CPT, and ICD‐10‐PCS Codes for HSCT Therapy.
**Table S6:** List of ICD‐9‐CM and ICD‐10‐CM codes for BTKi‐related malignancies and metastatic solid tumor.
**Table S7:** List of ICD‐9‐CM and ICD‐10‐CM codes for cardiac BTKi‐related events of interest.
**Table S8:** Occurrence of incident CVAEs while being treated with cBTKi stratified by LOT status.

## Data Availability

Data for this analysis was made available to the authors through a third‐party license from Optum CDM, a commercial data provider in the United States. As such, the authors cannot make these data publicly available due to data use agreement. Other researchers can access the data by purchasing a license through Optum CDM.
