# Supplementary material for: Healthcare Resource Utilization and Costs in Patients With Chronic Lymphocytic Leukemia or Small Lymphocytic Leukemia Treated With Covalent Bruton's Tyrosine Kinase Inhibitors: Real‐World Impact of Cardiovascular Adverse Events
Source: Cancer Med. 2025 Dec 21;14(24):e71484. doi: 10.1002/cam4.71484 (PMC12719235; doi:10.1002/cam4.71484)
Supplement: Supplementary file 1 — Table S1: List of ICD‐9‐CM and ICD‐10‐CM codes for CLL and SLL. Table S2: List of anticancer systemic agents CLL‐ or SLL‐related. Table S3: List of CPT codes for radiation therapies for CLL or SLL. Table S4: List of HCPCS, CPT, and ICD‐10‐PCS Codes for CAR‐T‐Cell Therapy. Table S5: List of HCPCS, CPT, and ICD‐10‐PCS Codes for HSCT Therapy. Table S6: List of ICD‐9‐CM and ICD‐10‐CM codes for BTKi‐related malignancies and metastatic solid tumor. Table S7: List of ICD‐9‐CM and ICD‐10‐CM codes for cardiac BTKi‐related events of interest. Table S8: Occurrence of incident CVAEs while being treated with cBTKi stratified by LOT status. [file CAM4-14-e71484-s001.docx]

**Supplementary Materials**

Supplemental Table 1. List of ICD-9-CM and ICD-10-CM Codes for CLL and SLL

| **Description** | **Diagnosis Code** |
| --- | --- |
| **CLL** |  |
| ***ICD-9-CM*** |  |
| Chronic lymphocytic leukemia, without mention of having achieved remission | 204.10 |
| Chronic lymphocytic leukemia, in remission | 204.11 |
| Chronic lymphocytic leukemia, in relapse | 204.12 |
| ***ICD-10-CM*** |  |
| Chronic lymphocytic leukemia of B-cell type not having achieved remission | C91.10 |
| Chronic lymphocytic leukemia of B-cell type in remission | C91.11 |
| Chronic lymphocytic leukemia of B-cell type in relapse | C91.12 |
| **SLL** |  |
| ***ICD-9-CM*** |  |
| Other named variants of lymphosarcoma and reticulosarcoma, unspecified site, extranodal and solid organ sites | 200.80 |
| Other named variants of lymphosarcoma and reticulosarcoma, lymph nodes of head, face, and neck | 200.81 |
| Other named variants of lymphosarcoma and reticulosarcoma, intrathoracic lymph nodes | 200.82 |
| Other named variants of lymphosarcoma and reticulosarcoma, intra-abdominal lymph nodes | 200.83 |
| Other named variants of lymphosarcoma and reticulosarcoma, lymph nodes of axilla and upper limb | 200.84 |
| Other named variants of lymphosarcoma and reticulosarcoma, lymph nodes of inguinal region and lower limb | 200.85 |
| Other named variants of lymphosarcoma and reticulosarcoma, intrapelvic lymph nodes | 200.86 |
| Other named variants of lymphosarcoma and reticulosarcoma, spleen | 200.87 |
| Other named variants of lymphosarcoma and reticulosarcoma, lymph nodes of multiple sites | 200.88 |
| ***ICD-10-CM*** |  |
| Small cell B-cell lymphoma, unspecified site | C83.00 |
| Small cell B-cell lymphoma, lymph nodes of head, face, and neck | C83.01 |
| Small cell B-cell lymphoma, intrathoracic lymph nodes | C83.02 |
| Small cell B-cell lymphoma, intra-abdominal lymph nodes | C83.03 |
| Small cell B-cell lymphoma, lymph nodes of axilla and upper limb | C83.04 |
| Small cell B-cell lymphoma, lymph nodes of inguinal region and lower limb | C83.05 |
| Small cell B-cell lymphoma, intrapelvic lymph nodes | C83.06 |
| Small cell B-cell lymphoma, spleen | C83.07 |
| Small cell B-cell lymphoma, lymph nodes of multiple sites | C83.08 |
| Small cell B-cell lymphoma, extranodal and solid organ sites | C83.09 |

**Supplemental Table 2. List of Anticancer Systemic Agents CLL- or SLL-Related**

| **Treatment** |
| --- |
| **Other chemo and targeted therapy combination** |
| Any combinations of at least one chemotherapy (see below) and one targeted therapy (see below, excluding monoclonal therapy) |
| **Chemotherapy** |
| Bendamustine |
| Chlorambucil |
| Cladribine |
| Cyclophosphamide |
| Fludarabine |
| Lenalidomide |
| Mechlorethamine |
| Pentostatin |
| **CIT** |
| Any combinations of at least one chemotherapy (see above) and one monoclonal antibody (see below) |
| **Targeted therapy** |
| **Kinase inhibitor** |
| *Bruton’s tyrosine kinase inhibitor* |
| Acalabrutinib |
| Ibrutinib |
| Zanubrutinib |
| *Phosphoinositide 3-kinase inhibitor* |
| Duvelisib |
| Idelalisib |
| **B-cell lymphoma-2 inhibitor** |
| Venetoclax |
| **CD20 monoclonal antibody** |
| Alemtuzumab |
| Obinutuzumab |
| Ofatumumab |
| Rituximab |
| Rituximab-abbs |

Abbreviations: CIT: chemoimmunotherapy

**Supplemental Table 3. List of CPT Codes for Radiation Therapies for CLL or SLL**

| **Description** | **CPT Code** |
| --- | --- |
| **2D** | 77280 |
|  | 77285 |
|  | 77290 |
| **3D Conformal** | 77295 |
| **Intensity Modulated Radiation Therapy** |  |
| Intensity modulated radiation treatment delivery; complex | 77386 |
| **Stereotactic Body Radiation Therapy** |  |
| Intensity modulated radiation therapy plan, including dose volume histogram | 77301 |
| Multi-leaf collimator device(s) for intensity modulated radiation therapy | 77338 |
| Stereotactic body radiation therapy, treatment delivery | 77373 |
| Stereotactic body radiation therapy, treatment management | 77435 |
| **Stereotactic Radiosurgery** |  |
| Special medical radiation physics consultation | 77370 |
| Radiation treatment delivery; multi-source Cobalt 60 based | 77371 |
| Radiation treatment delivery; linear accelerator based | 77372 |
| Stereotactic radiation treatment management of cranial lesion(s) | 77432 |

Abbreviations: 2D: two-dimensional; 3D: three-dimensional; CLL: chronic lymphocytic leukemia; CPT: Current Procedural Terminology; SLL: small lymphocytic lymphoma

**Supplemental Table 4. List of HCPCS, CPT, and ICD-10-PCS Codes for CAR-T-Cell Therapy**

| **Description** | **Code** |
| --- | --- |
| **HCPCS** |  |
| Adoptive immunotherapy i.e. development of specific anti-tumor reactivity (e.g., tumor-infiltrating lymphocyte therapy) per course of treatment | S2107 |
| Tisagenlecleucel, up to 250 million car-positive viable t cells, including leukapheresis and dose preparation procedures, per infusion | Q2040 |
| Axicabtagene Ciloleucel, up to 200 million autologous anti-CD19 CAR T cells, including leukapheresis and dose preparation procedures, per Infusion | Q2041 |
| Tisagenlecleucel, up to 600 million car-positive viable t cells, including leukapheresis and dose preparation procedures, per therapeutic dose. | Q2042 |
| **CPT** |  |
|  |  |
| Chimeric antigen receptor T-cell (CAR-T) therapy; harvesting of blood-derived T lymphocytes for development of genetically modified autologous CAR T-cells, per day | 0537T |
| Chimeric antigen receptor T-cell (CAR-T) therapy; preparation of blood-derived T lymphocytes for transportation (e.g. cryopreservation, storage) | 0538T |
| Chimeric antigen receptor T-cell (CAR-T) therapy; receipt and preparation of CAR T-cells for administration | 0539T |
| **ICD-10-PCS** |  |
| Introduction of Engineered Autologous Chimeric Antigen Receptor T-cell Immunotherapy into Peripheral Vein, Percutaneous Approach, New Technology Group 3 | XW033C3 |
| Introduction of Engineered Autologous Chimeric Antigen Receptor T-cell Immunotherapy into Central Vein, Percutaneous Approach, New Technology Group 3 | XW043C3 |

Abbreviations: CAR-T-cell: chimeric antigen receptor T cells; CPT: Current Procedural Terminology; HCPCS: Healthcare Common Procedure Coding System; ICD-10-PCS: International Classification of Diseases, 10th Revision, Procedure Coding System

**Supplemental Table 5. List of HCPCS, CPT, and ICD-10-PCS Codes for HSCT Therapy**

| **Description** | **Code** |
| --- | --- |
| **HCPCS** |  |
| Cord blood harvesting for transplantation, allogeneic | S2140 |
| Cord blood-derived stem-cell transplantation, allogeneic | S2142 |
| Bone marrow or blood-derived stem cells (peripheral or umbilical), allogeneic or autologous, harvesting, transplantation, and related complications; including: pheresis and cell preparation/storage; marrow ablative therapy; drugs, supplies, hospitalization with outpatient follow-up; medical/surgical, diagnostic, emergency, and rehabilitative services; and the number of days of pre-and post-transplant care in the global definition | S2150 |
| **CPT** |  |
| Blood-derived hematopoietic progenitor cell harvesting for transplantation, per collection; allogeneic | 38205 |
| Transplant preparation of hematopoietic progenitor cells; cryopreservation and storage | 38207 |
| Transplant preparation of hematopoietic progenitor cells; thawing of previously frozen harvest, without washing, per donor | 38208 |
| Transplant preparation of hematopoietic progenitor cells; thawing of previously frozen harvest, with washing, per donor | 38209 |
| Transplant preparation of hematopoietic progenitor cells; specific cell depletion within harvest, T-cell depletion | 38210 |
| Transplant preparation of hematopoietic progenitor cells; tumor cell depletion | 38211 |
| Transplant preparation of hematopoietic progenitor cells; red blood cell removal | 38212 |
| Transplant preparation of hematopoietic progenitor cells; platelet depletion | 38213 |
| Transplant preparation of hematopoietic progenitor cells; plasma (volume) depletion | 38214 |
| Transplant preparation of hematopoietic progenitor cells; cell concentration in plasma, mononuclear, or buffy coat layer | 38215 |
| Bone marrow harvesting for transplantation; allogeneic | 38230 |
| Hematopoietic progenitor cell (HPC); allogeneic transplantation per donor | 38240 |
| Hemic & Lymphatic - Bone marrow or blood-derived peripheral stem cell transplantation; autologous | 38241 |
| Allogeneic lymphocyte infusions | 38242 |
| HPC; HPC boost | 38243 |
| **ICD-10-PCS** |  |
| Transfusion of Embryonic Stem Cells into Peripheral Vein, Open Approach | 30230AZ |
| Transfuse Allo Rel Bone Marrow in Periph Vein, Open | 30230G2 |
| Transfuse Allo Unr Bone Marrow in Periph Vein, Open | 30230G3 |
| Transfuse Allo Unsp Bone Marrow in Periph Vein, Open | 30230G4 |
| Transfusion of Allogeneic Related Cord Blood Stem Cells into Peripheral Vein, Open Approach | 30230X2 |
| Transfusion of Allogeneic Unrelated Cord Blood Stem Cells into Peripheral Vein, Open Approach | 30230X3 |
| Transfusion of Allogeneic Unspecified Cord Blood Stem Cells into Peripheral Vein, Open Approach | 30230X4 |
| Transfusion of Autologous Hematopoietic Stem Cells into Peripheral Vein, Open Approach | 30230Y0 |
| Transfusion of Allogeneic Related Hematopoietic Stem Cells into Peripheral Vein, Open Approach | 30230Y2 |
| Transfusion of Allogeneic Unrelated Hematopoietic Stem Cells into Peripheral Vein, Open Approach | 30230Y3 |
| Transfusion of Allogeneic Unspecified Hematopoietic Stem Cells into Peripheral Vein, Open Approach | 30230Y4 |
| Transfusion of Embryonic Stem Cells into Peripheral Vein, Percutaneous Approach | 30233AZ |
| Transfuse Allo Rel Bone Marrow in Periph Vein, Perc | 30233G2 |
| Transfuse Allo Unr Bone Marrow in Periph Vein, Perc | 30233G3 |
| Transfuse Allo Unsp Bone Marrow in Periph Vein, Perc | 30233G4 |
| Transfusion of Allogeneic Related Cord Blood Stem Cells into Peripheral Vein, Percutaneous Approach | 30233X2 |
| Transfusion of Allogeneic Unrelated Cord Blood Stem Cells into Peripheral Vein, Percutaneous Approach | 30233X3 |
| Transfusion of Allogeneic Unspecified Cord Blood Stem Cells into Peripheral Vein, Percutaneous Approach | 30233X4 |
| Transfusion of Allogeneic Related Hematopoietic Stem Cells into Peripheral Vein, Percutaneous Approach | 30233Y2 |
| Transfusion of Allogeneic Unrelated Hematopoietic Stem Cells into Peripheral Vein, Percutaneous Approach | 30233Y3 |
| Transfusion of Allogeneic Unspecified Hematopoietic Stem Cells into Peripheral Vein, Percutaneous Approach | 30233Y4 |
| Transfusion of Embryonic Stem Cells into Central Vein, Open Approach | 30240AZ |
| Transfuse Allo Rel Bone Marrow in Central Vein, Open | 30240G2 |
| Transfuse Allo Unr Bone Marrow in Central Vein, Open | 30240G3 |
| Transfuse Allo Unsp Bone Marrow in Central Vein, Open | 30240G4 |
| Transfusion of Allogeneic Related Cord Blood Stem Cells into Central Vein, Open Approach | 30240X2 |
| Transfusion of Allogeneic Unrelated Cord Blood Stem Cells into Central Vein, Open Approach | 30240X3 |
| Transfusion of Allogeneic Unspecified Cord Blood Stem Cells into Central Vein, Open Approach | 30240X4 |
| Transfusion of Allogeneic Related Hematopoietic Stem Cells into Central Vein, Open Approach | 30240Y2 |
| Transfusion of Allogeneic Unrelated Hematopoietic Stem Cells into Central Vein, Open Approach | 30240Y3 |
| Transfusion of Allogeneic Unspecified Hematopoietic Stem Cells into Central Vein, Open Approach | 30240Y4 |
| Transfusion of Embryonic Stem Cells into Central Vein, Percutaneous Approach | 30243AZ |
| Transfuse Allo Rel Bone Marrow in Central Vein, Perc | 30243G2 |
| Transfuse Allo Unr Bone Marrow in Central Vein, Perc | 30243G3 |
| Transfuse Allo Unsp Bone Marrow in Central Vein, Perc | 30243G4 |
| Transfusion of Allogeneic Related Cord Blood Stem Cells into Central Vein, Percutaneous Approach | 30243X2 |
| Transfusion of Allogeneic Unrelated Cord Blood Stem Cells into Central Vein, Percutaneous Approach | 30243X3 |
| Transfusion of Allogeneic Unspecified Cord Blood Stem Cells into Central Vein, Percutaneous Approach | 30243X4 |
| Transfusion of Allogeneic Related Hematopoietic Stem Cells into Central Vein, Percutaneous Approach | 30243Y2 |
| Transfusion of Allogeneic Unrelated Hematopoietic Stem Cells into Central Vein, Percutaneous Approach | 30243Y3 |
| Transfusion of Allogeneic Unspecified Hematopoietic Stem Cells into Central Vein, Percutaneous Approach | 30243Y4 |
| Transfusion of Nonautologous Bone Marrow into Peripheral Artery, Open Approach | 30250G1 |
| Transfusion of Nonautologous Bone Marrow into Peripheral Artery, Percutaneous Approach | 30253G1 |
| Transfusion of Nonautologous Bone Marrow into Central Artery, Open Approach | 30260G1 |
| Transfusion of Nonautologous Bone Marrow into Central Artery, Percutaneous Approach | 30263G1 |

Abbreviations: CLL: chronic lymphocytic leukemia; CPT: Current Procedural Terminology; HCPCS: Healthcare Common Procedure Coding System; HSCT: hematopoietic stem cell transplantation; ICD-10-PCS: International Classification of Diseases, 10th Revision, Procedure Coding System

**Supplemental Table 6. List of ICD-9-CM and ICD-10-CM Codes for BTKi-Related Malignancies and Metastatic Solid Tumor**

| **Condition** | **ICD-9-CM** | **ICD-10-CM** |
| --- | --- | --- |
| Metastatic solid tumor | 196.x-199.x | C77.x-C80.x |
| Chronic graft versus host disease | 279.52 | D89.811 |
| CLL or SLL | See Supplementary Table 1 | |
| Mantle cell lymphoma | 200.4x | C83.1x |
| Marginal zone lymphoma | 200.3x | C88.4 |
| Waldenstrom's macroglobulinemia | 273.3 | C88.0 |

**Abbreviations**: BTKi: Bruton’s tyrosine kinase inhibitor; CLL: chronic lymphocytic leukemia; SLL: small lymphocytic lymphoma

**Supplemental Table 7. List of ICD-9-CM and ICD-10-CM Codes for Cardiac BTKi-Related Events of Interest**

| **Condition** | **ICD-9-CM** | **ICD-10-CM** |
| --- | --- | --- |
| Atrial fibrillation | 427.31 | I48.0-I48.2, I48.91 |
| Typical and atypical atrial flutter | 427.32 | I48.3, I48.4 |
| Unspecified atrial flutter |  | I48.9 |
| Heart failure | 398.91, 425.4x-425.5x, 425.7x-425.9x, 428.xx | I09.9, I11.0, I13.0, I13.2, I25.5, I42.0, I42.5-I42.9, I43.x, I50.x, P29.0 |
| Ventricular arrhythmia | 427.1, 427.4 | I47.0, I47.2, I49.0 |
| Ventricular premature depolarization |  | I49.3 |
| Hypertension | 401-405 | I10-I13, I15, I16 |
| Conduction disorders | 426.9 | I45.8-I45.9 |
| Cardiomyopathy | 425.0, 425.11, 425.18, 425.2, 425.4, 425.5, 425.9 | I42.x, I43.x |
| Myocardial infarction | 410.x | I21.x, I22.x, I25.2 |
| Cardiac-related death | 427.5, 798, 798.1, 798.2 | I46.9, R99 |

**Supplemental Table 8. Occurrence of incident CVAEs while being treated with cBTKi stratified by LOT status**

| **CVAE**^†^ | **1L  (N=1,691)** | **2L+  (N=378)** |
| --- | --- | --- |
| **Atrial fibrillation** | N = 1,452 | N = 298 |
| Occurrence, n (%) | 134 (9.2%) | 37 (12.4%) |
| Occurrence rate, PPPM | 8.3 | 12.0 |
| **Atrial flutter** | N = 1,483 | N = 301 |
| Occurrence, n (%) | 128 (8.6%) | 36 (12.0%) |
| *Typical/Atypical* | *55 (3.7%)* | *17 (5.7%)* |
| *Unspecified atrial flutter only* | *73 (4.9%)* | *19 (6.3%)* |
| Occurrence rate, PPPM | 7.7 | 11.5 |
| **Heart failure** | N = 1,385 | N = 278 |
| Occurrence, n (%) | 116 (8.4%) | 17 (6.1%) |
| Occurrence rate, PPPM | 7.4 | 5.8 |
| **Ventricular arrhythmias** | N = 1,335 | N = 268 |
| Occurrence, n (%) | 129 (9.7%) | 25 (9.3%) |
| *Non-PVC-related* | *32 (2.4%)* | *10 (3.7%)* |
| *PVC-only* | *97 (7.3%)* | *15 (5.6%)* |
| Occurrence rate, PPPM | 8.5 | 9.0 |
| **Hypertension** | N = 366 | N = 78 |
| Occurrence, n (%) | 81 (22.1%) | 14 (17.9%) |
| Occurrence rate, PPPM | 21.8 | 20.3 |
| **Cardiac-related death** | N = 1,667 | N = 366 |
| Occurrence, n (%) | 18 (1.1%) | 6 (1.6%) |
| Occurrence rate, PPPM | 0.9 | 1.6 |
| **Conduction disorders** | N = 1,659 | N = 367 |
| Occurrence, n (%) | 8 (0.5%) | <5 (<1%) |
| Occurrence rate, PPPM | 0.4 | 0.0 |
| **Cardiomyopathy** | N = 1,596 | N = 355 |
| Occurrence, n (%) | 43 (2.7%) | 5 (1.4%) |
| Occurrence rate, PPPM | 2.3 | 1.4 |
| **Myocardial infarction** | N = 1,506 | N = 328 |
| Occurrence, n (%) | 57 (3.8%) | 9 (2.7%) |
| Occurrence rate, PPPM | 3.3 | 2.6 |

^†^ The percentage for each CVAE occurrence is calculated relative to the total number of patients who did not have that specific CVAE in their entire medical history within the Optum^®^ CDM database.

**Abbreviations:** LOT, Line of therapy; 1L, First-line therapy observed; 2L, Second-line therapy observed; CVAE, Cardiovascular Adverse Event; PPPM, Per 1000 Patient Months; PVC, Premature Ventricular Contraction
